# Relationship between indication for tooth extraction and outcome of immediate implants: A retrospective study with 5 years of follow-up

**DOI:** 10.4317/jced.51616

**Published:** 2014-10-01

**Authors:** Beatriz Tarazona, Pablo Tarazona-Álvarez, David Peñarrocha-Oltra, Maria Peñarrocha-Diago

**Affiliations:** 1 Associate Professor of Orthodontics. Department of Stomatology, Valencia University Medical and Dental School, Spain; 2 Master of Oral Surgery and Implantology. Department of Stomatology, Valencia University Medical and Dental School, Spain; 3 Full Professor of Oral Surgery, Department of Stomatology, Valencia University Medical and Dental School, Spain

## Abstract

Objectives: The aims of this retrospective study were to evaluate the survival rate of a series of immediate implants after 3 years of follow-up and to study the relationship between survival and indication for tooth extraction. Study Design: A retrospective study of patients treated with immediate implants between January 2003 and December 2008 was carried out. All patients receiving at least one post-extraction implant and a minimum follow-up of 5 years were included. Results: After 60 months, 30 immediate implants had been lost in 17 patients, yielding a total implant success rate of 93.8%. None of the implants placed failed after the extraction of included canines (100% success rate). In 20 failed implants the reason for extraction had been severe periodontal disease (91.8% SR), in 4 endodontic failure (88.6%SR), in 3 unrestorable caries (95.9% SR), in 1 untreatable fracture (95.2% SR) and in 2 improvement of prosthetic design (98.1% SR). No statistically significant influence was found between immediate implant failure and the reason for tooth extraction (p=0.11). Conclusions: The use of immediate implants is a successful alternative to replace missing teeth for severe periodontal disease, periapical pathology or by decay or untreatable fractures. Some reasons, such as periodontal disease itself is associated with a success rate significantly below the overall average. Similarly, the prosthetic design is associated with a better prognosis than all other reasons.

** Key words:**Tooth extraction, immediate implants, success rate.

## Introduction

The placement of implants in fresh extraction sockets was first described by Schulte & Heimke (1), who referred to this procedure as ‘immediate implant’. In contrast, implant placement in healed sites is known as conventional, delayed or non-immediate procedure. Delayed dental implant placement has since long been used to replace lost teeth due to different reasons, including periodontal disease, failure of endodontic treatment and untreatable fractures or caries.

According to the literature, implants placed following a delayed procedure after waiting for the healing of sockets of infected teeth have similar success rates than those replacing healthy teeth (2). Delayed implants are also a common alternative to replace teeth absent due to agenesis or included (3).

Several advantages have been associated with immediate implant placement, including reduced overall treatment time, reduced number of surgical procedures, better esthetic outcome and, thus, high patient acceptance (4). Therefore, immediate implants should be considered an alternative to replace teeth extracted both due to pathology –severe periodontal disease, endodontic failure, periapical pathology, fracture or untreatable caries- and to improve prosthetic design (5-7).

 Tooth infection is considered a relative contraindication for immediate implant placement, although some case series indicate that results may be similar as with implants placed in healthy sockets (8). In some cases immediate implants may also be indicated simultaneous to the extraction of included teeth (9).

Immediate implants are nowadays a well-accepted procedure that has been shown to have high success rates in studies with large samples and long follow-up periods (10,11). However, some teeth pathologies or conditions which constitute an indication for extraction could influence the prognosis of immediately placed implants. High success rates achieved by dental implants make necessary large samples to statistically study relationships between failure and variables such as indication for tooth extraction; thus, evidence in the literature on this topic is limited.

The aims of this retrospective study were to evaluate the survival rate of a series of immediate implants after 5 years of follow-up and to study the relationship between survival and indication for tooth extraction.

## Material and Methods

- Patient selection

A retrospective study of patients treated with immediate implants between January 2003 and December 2008 was carried out. All patients receiving at least one post-extraction implant and a minimum follow-up of 5 years were included. Patients with missing data or incomplete protocols were excluded.

The intervention was not performed in patients with systemic or psychological disorders that contraindicate oral surgery, and immediate implants were not placed in the presence of acute periapical or periodontal infection, or fistula in the soft tissues.

This research was performed following the principles of the Declaration of Helsinki regarding research on humans; signature of a written informed consent form from all patients was requested. As data were retrospectively collected, approval by an ethical board was not necessary.

- Surgical procedure

All surgeries were performed by the same surgeon (MPD), under local anesthesia (4% articaine with 1:100,000 adrenalin (Inibsa ®, Lliça of Vall, Barcelona, Spain). In complex cases, involving the extraction of several teeth and the placement of a large number of implants, intravenous conscious sedation was performed with 1% propofol solution (Diprivan ®, Astra Zeneca Pharma SA, Madrid, Spain) administered by an anesthesiologist. To allow immediate implant placement, extraction of teeth was done with great care; in multiradicular teeth odontosection was performed and roots were extracted separately, in order to respect the alveolar walls.

A thorough curettage of the socket was performed to remove any infected or inflamed tissue and remnants of the periodontal ligament. In antero-superior teeth implants were placed palatally; in upper molars and premolars with two roots they were placed in the palatine root. In all cases drills and osteotomes were combined to carve the implant beds. In the mandibular posterior area implants were placed, whenever possible, in the interradicular septum. Antero-inferior implants were placed as parallel as possible.

In cases of fenestration or dehiscence of the buccal wall that left exposed threads, or when the gap was greater than 2 mm, particulate autologous bone was used alone if enough had been collected during the surgery, or mixed with ß-tricalcium phosphate (KeraOs®, Keramat, Santiago de Compostela, Spain). In large fenestrations or dehiscences, leaving more than 3-4 threads exposed, a resorbable collagen membrane (Lyostypt®, B. Braun, Aesculap, Tuttlingen, Germany) was used to cover the particulate bone graft. Autologous bone was obtained from the implantation area, the maxillary tuberosity, the retromolar region or the chin.

Implants were left submerged whenever bone grafting techniques had been used, and in patients wearing temporary removable prosthesis. A postoperative panoramic radiography was done in all cases.

Antibiotics were prescribed (Clamoxyl, GlaxoSmithKline; 500mg three times a day for 7 days), together with ibuprofen (Bexistar, Laboratorio Bacino; 600mg three times a day for 3 days) and 0.12% chlorhexidine rinse (GUM, John O Butler/Sunstar). Sutures were removed 7 days after the surgery. The second surgery was performed after 8 weeks in the maxilla and after 6 weeks in the mandible; prosthetic loading, after 10 weeks in the maxilla and 8 weeks in the mandible.

- Data gathering and follow-up

The following variables were recorded: patient age (at implant placement) and sex (male/female); implant length, diameter and position; widest horizontal gap between the implant and socket walls; condition indicative of tooth extraction (tooth inclusion, severe periodontal disease, endodontic failure, unrestorable caries, untreata-ble fracture, or prosthetic design); and type of final prosthesis.

Clinical and radiographic assessment was performed at implant loading and every 12 months thereafter. Radiographic assessment was done with panoramic radiographs (digital orthopantomograph OP100, Instrumentarium Imaging, Tuusula, Finland), which were used to not to calculate an exact peri-implant bone loss value but to identify severe loss (>3 mm after 5 years of follow-up).

- Statistical Analysis

To test the hypothesis of homogeneity of the success rate for different reasons of extraction the Chi2 test was applied.

The Chi2 test was used with a significance level of 5% and considering an effect size of 0.2 (small-medium), reached a power of 93.3% in a sample of 487 cases.

## Results

**Figure 1:**
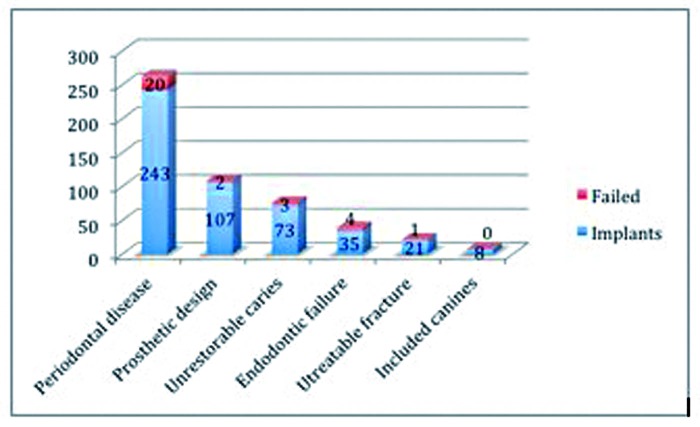
Details the number of immediate implants failed placed with respect to the principal reason for teeth extraction.

Four hundred and ninety-six immediate implants were used to substitute extracted teeth in 167 patients that fulfilled the inclusion criteria; four patients with nine implants were excluded due to missing data. The study sample thus comprised 157 patients (74 males and 83 females) with a mean age of 55,4 years (range 25 to 80 years) that received four hundred and eighty seven immediate implants, 290 were placed in the maxilla and 197 in the jaw, and were followed for at least 5 years. Immediate implants were placed after the extraction of 8 included canines, 238 teeth with severe periodontal disease, 58 with endodontic failure, 75 with unrestorable caries, 20 with untreatable fracture and 88 healthy teeth extracted for prosthetic reasons (Fig. 1).

The most common implant lengths were 11.5 mm (21.1%) and 13 mm (31.6%); 64% of the implants had a diameter of 4.2 mm, 28% of 5.5 mm and 8% of 3.3 mm. 112 immediate implants were placed in patients that smoked, and in 160 the gap was wider than 2 mm; autologous bone obtained during drilling was used to fill it in alone in 78 implants and combined with ß-tricalcium phosphate (KeraOs®) in 82. 43 implants were rehabilitated with single crowns, 197 with partial-arch fixed prostheses, 149 with full-arch fixed prostheses and 70 with over-dentures. No statistically significant relation was found between implant outcome and implant length or diameter, smoking, gap filling or type of prosthesis. (*p*=0,65)

After 60 months, 30 immediate implants had been lost in 17 patients, yielding a total implant success rate of 93.8%. None of the implants placed failed after the extraction of included canines (100% success rate). In 20 failed implants the reason for extraction had been severe periodontal disease (91.8% SR), in 4 endodontic failure (88.6%SR), in 3 unrestorable caries (95.9% SR), in 1 untreatable fracture (95.2% SR) and in 2 improvement of prosthetic design (98.1% SR). No statistically significant influence was found between immediate implant failure and the reason for tooth extraction (*p*=0.11).

Twenty were early (before prosthetic loading) and 10 late failures (after prosthetic loading).  Table 1 details the characteristics of the failed implants, which 19 implants were placed in the posterior and 11 were placed in the front. Of the remaining 457 implants none had presented with pain, infection, bleeding, mobility or periimplant radiolucency up to the last control visit.

**Table 1:**
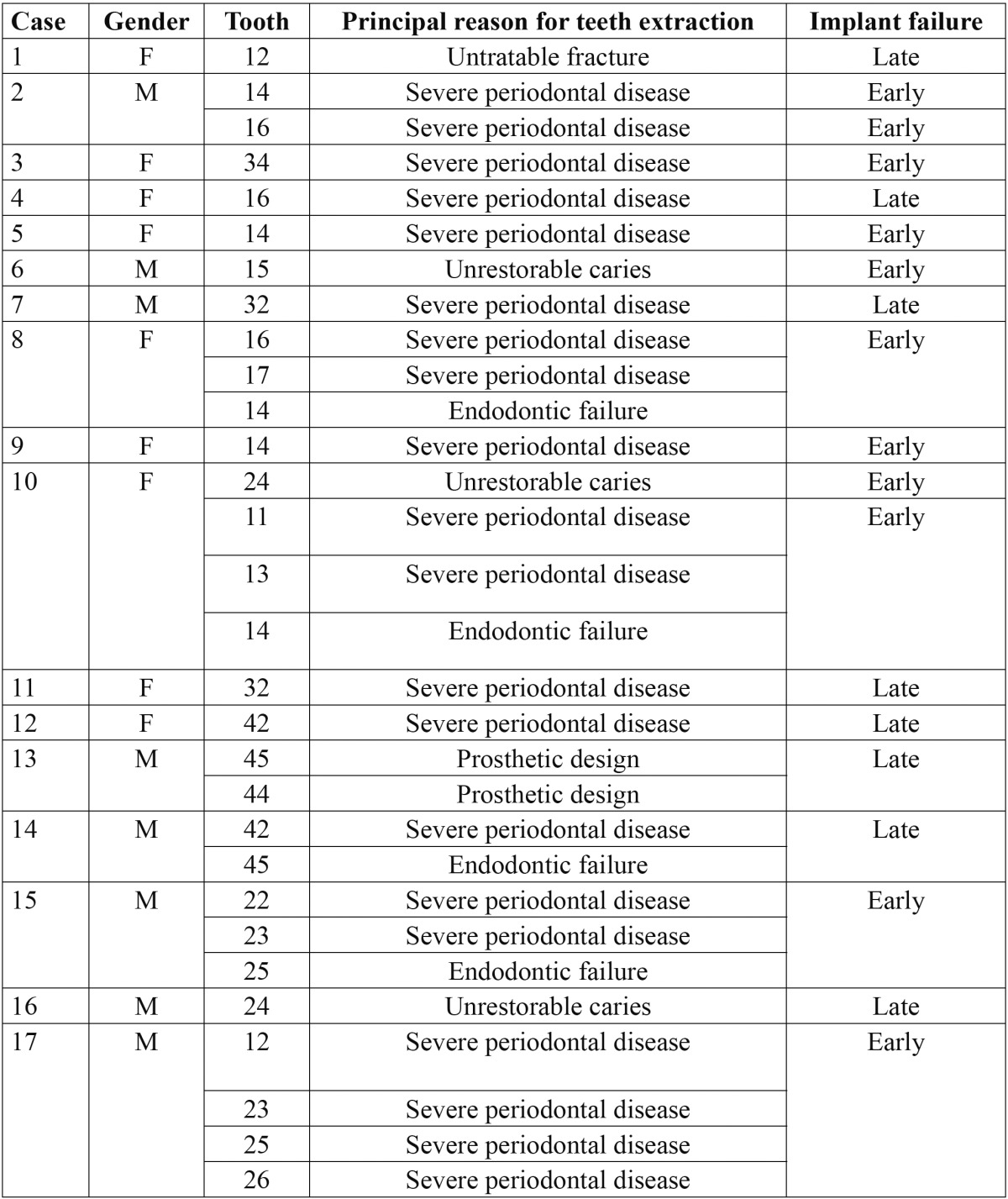
Relationship between reason for teeth extraction and failed implant.

## Discussion

The review of the literature yielded favorable results with implants placed immediately after the extraction of included teeth. Garcia *et al.* (9) placed 10 immediate implants after removing included upper canines. The canines were carefully removed to preserve the bone crest, and the implants were placed with bicortical anchorage, achieving all primary stability. All cases were grafted with particulate autologous bone obtained during drilling. One year after loading the success rate was 100% and the average peri-implant marginal bone loss was 0.49 mm. In this study 8 implants were placed after the extraction of included maxillary teeth and a 100% success rate was obtained for these implants.

Alves *et al.* (12) placed 168 implants (108 immediate implants) in 23 periodonatally compromised patients; only 2 non-immediate implants did not osseointegrate, yielding an overall 3-year cumulative survival rate of 98.74% (100% for immediately placed implants). Maló *et al.* (13) placed a total of 68 immediate implants in sockets of periodontally compromised teeth divided into 2 groups: in the retrospective group (31 implants) 4 immediate implants failed (survival rate of 87.1%) and the average bone loss after 1 year was 1.6 mm; in the prospective group (37 implants) the authors used a postsurgical protocol to control inflammation (ruled prednisone 5 mg and ibuprofen 600 mg) and a standard maintenance (hyaluronic acid gel during the first 2 months and clorhexidine gel during the next 2), and achieved a survival rate of 100% and an average bone loss of 0.8 mm.

Ferrus *et al.*(14) studied factors that could possibly influence ridge alterations following immediate implant placement into extraction sockets; one of the factors studied was if the cause of tooth extraction was periodontitis or not. They placed 93 single-tooth immediate implants in 93 subjects, and their results suggested that the presence or not of periodontal disease in extracted teeth had no influence in the amount of hard tissue alteration during the first 4 months after placing immediate implants. Similarly, in this study 248 immediate implants were placed after removing teeth with severe periodontal disease; 17 of these implants failed, yielding a success rate of 93.1%, and no statistically significant difference was found between either success or marginal bone loss of these implants and those of the control group (placed in sockets of healthy teeth).

Several authors have considered chronic periodontal disease a risk factor for implant failure, regardless of the time of placement with respect to the dental extraction (12,13,15,16).

Chen and Buser (17) in their review found that for many authors bone augmentation procedures are effective in promoting bone fill and defect resolution at implants in postextraction sites, and are more successful with immediate and early placement than with late placement. The majority of studies reported survival rates of over 95%. Similar survival rates were observed for immediate and early placement.

Several authors have placed implants in fresh-sockets of teeth with periapical infections. Crespi *et al.* (18) placed 30 immediate implants (15 in teeth without periapical lesions and 15 in teeth with periapical lesions but no pain or fistula); at the 24-month follow-up the survival rate of both groups was 100% and no statistically significant differences existed in bone loss. Similarly, Truninger *et al.* (19) placed 13 immediate implants in patients with periapical pathology and 16 in patients without pathology; after 3 years all implants still survived and radiographic evaluation revealed no differences in bone loss. Lindeboom *et al.* (20) randomly placed 25 immediate or 25 delayed implants in 50 patients with chronic periapical infection. Bone regeneration was performed in immediate implants with particulate bone graft and membranes. Only 2 implants failed in the immediate group, resulting in respective survival rates of 92% and 100%, and no statistically significant differences were found between both groups with respecto to ISQ values, radiographic bone loss and gingival esthetics. In this study 31 implants were placed immediately after the extraction of teeth with chronic periapical pathology and 0 failed; their success rate was 100% and their average bone loss 0,54 mm, existing no statiscally significant differences between these values and the average media of the study. Furthermore, in the present study, as in all of the studies reviewed, thorough socket debridement and systemic antibiotics were used.

Villa & Rangert (21) studied the immediate placement of implants to substitute teeth with infection: they placed 76 implant -55 in sockets of teeth with periodontal disease, 15 with chronic periapical infection and 6 with root fracture- which were immediately loaded with fixed prostheses. During the first year 2 implants were lost, both place in sites with periodontal disease, resulting in an overall 97.4% survival rate, and a mean marginal bone loss of 0.91 mm. In the present study 92 immediate implants were used to replace non-restorable teeth due to fracture or caries, with a success rate of 95.2% and 95.9%.

## Conclusions

The use of immediate implants is a successful alternative to replace missing teeth for severe periodontal disease, periapical pathology or by decay or untreatable fractures. Some reasons, such as periodontal disease itself is associated with a success rate significantly below the overall average. Similarly, the prosthetic design is associated with a better prognosis than all other reasons.
